# Phosphorus Modification of Iron: Mechanistic Insights into Ammonia Synthesis on Fe_2_P Catalyst

**DOI:** 10.3390/molecules29081894

**Published:** 2024-04-22

**Authors:** Abdulrahman Almithn

**Affiliations:** Department of Chemical Engineering, College of Engineering, King Faisal University, Al Ahsa 31982, Saudi Arabia; aalmithn@kfu.edu.sa

**Keywords:** iron phosphides, ammonia synthesis, density functional theory, N_2_ dissociation, reaction mechanism

## Abstract

Ammonia (NH_3_) is a critical chemical for fertilizer production and a potential future energy carrier within a sustainable hydrogen economy. The industrial Haber–Bosch process, though effective, operates under harsh conditions due to the high thermodynamic stability of the nitrogen molecule (N_2_). This motivates the search for alternative catalysts that facilitate ammonia synthesis at milder temperatures and pressures. Theoretical and experimental studies suggest that circumventing the trade-off between N–N activation and subsequent NH*_x_* hydrogenation, governed by the Brønsted–Evans–Polanyi (BEP) relationship, is key to achieving this goal. Recent studies indicate metal phosphides as promising catalyst materials. In this work, a comprehensive density functional theory (DFT) study comparing the mechanisms and potential reaction pathways for ammonia synthesis on Fe(110) and Fe_2_P(001) is presented. The results reveal substantial differences in the adsorption strengths of NH*_x_* intermediates, with Fe_2_P(001) exhibiting weaker binding compared to Fe(110). For N–N bond cleavage, multiple competing pathways become viable on Fe_2_P(001), including routes involving the pre-hydrogenation of adsorbed N_2_ (e.g., through *NNH*). Analysis of DFT-derived turnover rates as a function of hydrogen pressure (H_2_) highlights the increased importance of these hydrogenated intermediates on Fe_2_P(001) compared to Fe(110) where direct N_2_ dissociation dominates. These findings suggest that phosphorus incorporation modifies the ammonia synthesis mechanism, offering alternative pathways that may circumvent the limitations of traditional transition metal catalysts. This work provides theoretical insights for the rational design of Fe-based catalysts and motivates further exploration of phosphide-based materials for sustainable ammonia production.

## 1. Introduction

Ammonia (NH_3_) is an indispensable chemical compound with vast applications in agriculture, supporting the production of fertilizers essential for global food security. Additionally, ammonia is increasingly recognized as a potential energy carrier within a future hydrogen economy, demonstrating its value in sustainable energy systems [1]. The Haber–Bosch process, developed over a century ago, remains the industrial mainstay of ammonia production. It relies on iron-based catalysts to convert atmospheric nitrogen (N_2_) and hydrogen (H_2_) to ammonia at high temperatures and pressures [2]. Despite its success, the Haber–Bosch process is notoriously energy-intensive, consuming approximately 2% of global energy production [3]. Consequently, driving ammonia synthesis under milder conditions is a long-standing challenge in catalysis with extensive economic and environmental implications.

The central obstacle to efficient, low-energy ammonia synthesis lies in the inert nature of the N_2_ molecule. The exceptionally strong nitrogen triple bond (N≡N) necessitates harsh reaction conditions to facilitate its dissociation, a key step in ammonia formation [4,5,6,7,8]. Furthermore, the surface intermediates formed during the reaction (NH*_x_*, *x* = 0–3) often bind strongly to transition metal catalysts, requiring sustained high temperatures and pressures to drive the reaction towards completion [9].

Extensive theoretical and experimental research has aimed to identify catalysts that effectively activate N_2_ while facilitating the sequential hydrogenation steps towards NH_3_. Density Functional Theory (DFT) calculations have revealed a key limitation in this pursuit: the Brønsted–Evans–Polanyi (BEP) scaling relationship. This relationship dictates that the nitrogen–nitrogen transition state energy is fundamentally linked to the binding energy of NH*_x_* intermediates [10,11,12,13,14]. Metals which readily cleave the N–N bond also bind NH*_x_* intermediates strongly, thus inhibiting NH_3_ product formation. As a result, traditional transition metal catalysts exhibit a volcano-shaped dependence of turnover frequency on N* binding energy, with Fe and Ru near the top [10]. Circumventing this limitation is essential for the design of more efficient catalysts.

To address this challenge, research has explored strategies such as combining metals on opposite sides of the volcano (e.g., Co–Mo catalysts) [15]. Moreover, studies have ventured beyond pure transition metals, investigating alternative materials with modified electronic structures. Metal nitrides, in particular transition metal nitrides, have shown promise for decoupling N_2_ activation and hydrogenation through the Mars–van Krevelen mechanism, potentially mitigating the BEP scaling relationship [16,17]. Additionally, hydrides and oxides have emerged as interesting candidates with potential benefits in ammonia synthesis [18,19].

Iron (Fe) and its compounds remain compelling catalysts for ammonia synthesis due to their abundance, established industrial use, and relative activity. Pioneering work by Somorjai et al. revealed the structure-sensitive nature of this reaction, demonstrating the varying activity of different iron crystal facets [20]. They found that the relative order of activity for ammonia formation was Fe(111) > Fe(100) > Fe(110) at high pressures and temperatures (20 atm and 638–723 K). The reaction mechanism proceeds through N_2_ dissociation, which is considered as the rate-limiting step, and is followed by sequential hydrogenation steps to form NH, NH_2_, and finally NH_3_ [4,21]. DFT studies have played a crucial role in furthering our understanding of the reaction mechanism, identifying preferred adsorption sites for the intermediates involved, and calculating the associated energy barriers. 

Recent studies have explored the potential of iron phosphides, particularly Fe_2_P, which exhibits modified surface electronic structures compared to pure iron, for the electrosynthesis of ammonia from nitrite [22,23]. Fe_2_P offers a departure from pure iron with its modified surface electronic structure. The addition of phosphorus may alter the interaction between the catalyst and nitrogen-containing intermediates, potentially influencing the reaction mechanism and ammonia synthesis activity. This change echoes previous research demonstrating the impact of phosphorus incorporation on transition metal catalysts’ properties. For example, nickel phosphide catalysts exhibit enhanced selectivity towards breaking tertiary ^3^C–O bonds in oxygenate compounds, in contrast to pure nickel catalysts where secondary ^2^C–O bond cleavage is favored [24]. Furthermore, phosphorus incorporation improved activity and selectivity in other reactions, such as methanol steam reforming and methane activation [25,26]. These findings suggest that phosphorus modification could potentially fine-tune catalytic properties for specific target reactions [27].

In this work, a comprehensive DFT study comparing the ammonia synthesis mechanisms on Fe and Fe_2_P catalysts is presented. These calculations aim to elucidate the key differences in N_2_ activation, NH*_x_* intermediate binding, and hydrogenation pathways on these two surfaces. Additionally, a strategy to weaken the N–N bond by pre-hydrogenation prior to cleavage is explored. For example, the findings of this study demonstrate that cleaving the N–N bond in the NNH_2_* intermediate is much more facile than direct N_2_ activation over Fe_2_P. By understanding these fundamental factors governing the catalytic performance of iron phosphide, the goal is to advance the rational design of improved iron-based catalysts for sustainable, low-temperature ammonia synthesis.

## 2. Results and Discussion

### 2.1. Optimized Adsorbates and Their Binding Energies

**Figure 1:**
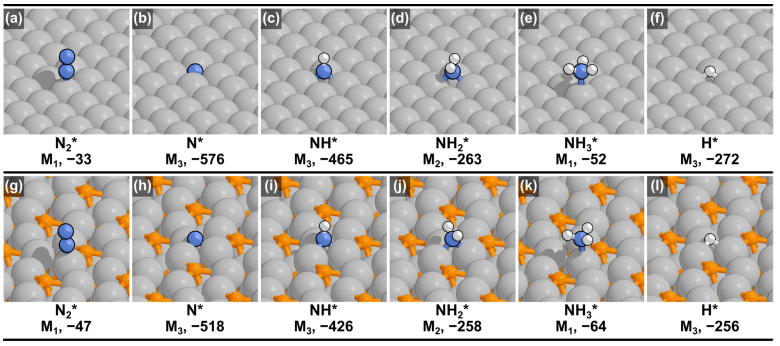
DFT-optimized structures for NH_x_* (*x* = 0–3) and H* on (**a**–**f**) Fe and (**g**–**l**) Fe_2_P. The binding energies (in kJ mol^−1^) and adsorption modes are shown beneath each image (see Section 3 for more details).

The adsorption of reactants, intermediates, and products on catalytic surfaces significantly impacts the mechanisms and feasibility of ammonia synthesis. Therefore, this section investigates the optimized geometries and binding energies for all relevant nitrogen-containing intermediates (N_2_, N, NH, NH_2_, NH_3_) and hydrogen on Fe and Fe_2_P surfaces. The goal is to identify potential differences in how these catalysts stabilize key intermediates, offering insights into potential variations in the reaction pathways. Figure 1 illustrates the adsorption configurations while Table 1 shows the associated binding energies (calculated using Equation (1) relative to gas-phase species and a clean catalyst surface). The BEP scaling relationship suggests that these energies directly influence the feasibility of subsequent hydrogenation steps [10,11,12,13,14]. Surfaces that readily break the N–N bond tend to bind NH*_x_* intermediates more strongly. This strong binding, however, can hinder the final hydrogenation steps necessary to form NH_3_. Analyzing the thermodynamic stability of these intermediates provides insights into the distinct steps potentially involved in the N_2_ dissociation and hydrogenation on each catalyst.

On the Fe(110) surface, the DFT calculations reveal a clear trend in the preferred adsorption sites and binding energies for the nitrogen-containing intermediates, where the interaction with the surface weakens as the species become more saturated with hydrogen (NH, NH_2_, NH_3_). N_2_ favors the atop site (M_1_) with a binding energy of −33 kJ mol^−1^ (Figure 1a), consistent with established theoretical expectations [28,29]. Atomic nitrogen (N*) and the NH* radical exhibit a strong preference for the three-fold hollow site (M_3_), with substantial binding energies of −576 and −465 kJ mol^−1^ (Figure 1b,c), respectively. This strong interaction aligns with previous adsorption studies on Fe(110) and Fe(111), a similarly close-packed surface [28,30]. NH_2_* prefers the bridge site (M_2_) and NH_3_* binds weakly atop a single Fe atom, indicating a favorable pathway towards desorption. Hydrogen (H*) occupies the three-fold site with a binding energy of −272 kJ mol^−1^, a finding corroborated by previous theoretical work on Fe surfaces [30]. 

It is important to acknowledge that the RPBE functional employed in this study does not account for attractive dispersion forces. These forces can significantly increase the binding energy, particularly for large physisorbed species and larger adsorbates in general. However, vdW-based methods that account for dispersion interactions are known to often overestimate the binding energy of smaller chemisorbed species. Including dispersion interactions here would likely shift the binding energies of chemisorbed species in a relatively consistent manner. Therefore, the relative energy barriers between the elementary steps investigated here would remain largely unchanged.

The Fe_2_P(001) catalyst surface exhibits notable differences in adsorption behavior compared to Fe(110) (Table 1). While N_2_ still weakly favors a metal atop site (M_1_, −47 kJ mol^−1^), its binding energy is slightly stronger on Fe_2_P (Figure 1g). More striking is the shift in binding strength for atomic nitrogen (N*) and NH* (Figure 1h,i). The presence of phosphorus weakens the interaction with these intermediates, with binding energies of −518 kJ mol^−1^ and −426 kJ mol^−1^ on Fe_2_P(001) compared to the substantially stronger binding on Fe(110). Similar to Fe(110), N* and NH* favor the three-fold hollow site (M_3_) while NH_2_* prefers the bridging site (M_2_). Ammonia (NH_3_*) continues to show weak binding in the atop position (M_1_). However, it binds slightly more strongly (by 12 kJ mol^−1^) on Fe_2_P compared to Fe. The adsorption of hydrogen (H*) on the three-fold site (M_3_) of Fe_2_P(001) aligns with observations on Fe(110), although the binding energy is slightly lower. 

These findings suggest that the presence of phosphorus in Fe_2_P weakens the binding strength of NH*_x_* species compared to Fe(100). The contrasting binding trends for N_2_ and NH*_x_* on Fe and Fe_2_P have potential implications for the reaction mechanisms. The weaker NH*_x_* binding on Fe_2_P could facilitate their hydrogenation steps compared to Fe, potentially leading to enhanced ammonia synthesis rates. Interestingly, this weakening does not extend to N_2_ or NH_3_, which show slightly increased binding energies on Fe_2_P. These observations can be understood by considering the electronic effects of phosphorus. Previous DFT studies on Ni_2_P catalysts for selective C–O bond cleavage suggest that the addition of phosphorus introduces Lewis acid sites [27,31]. This Lewis acidity arises from a small charge transfer from the transition metal (Fe in our case) to phosphorus. NH_3_, being a Lewis base, interacts more favorably with the Lewis acid sites on metal phosphides, as demonstrated previously for Ni_2_P compared to pure Ni. 

### 2.2. N–N Bond Activation Pathways on Fe(110)

**Scheme 1:**
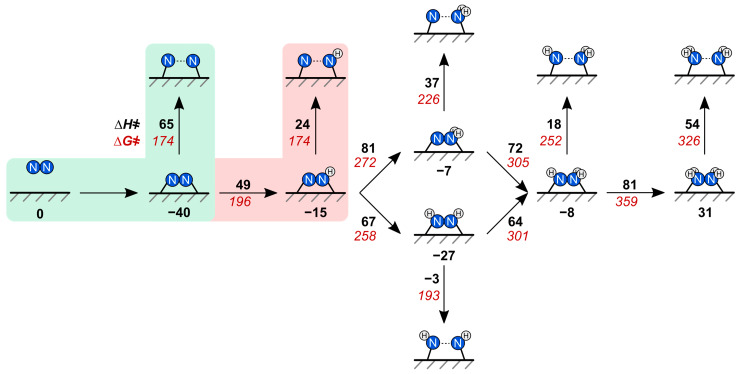
The examined reactions network on Fe(110) surface. Effective enthalpy (Δ*H*҂) and free energy (Δ*G*҂) barriers are shown in kJ mol^−1^ at 673 K, calculated relative to gas-phase N_2_ and H_2_ using Equations (2) and (3).

Understanding the mechanism and energetics of N–N bond activation is crucial for designing effective ammonia synthesis catalysts since this step is often considered rate-limiting. This section analyzes the calculated N–N bond dissociation barriers on Fe(110) for both molecular N_2_ and its hydrogenated intermediates (N_2_H*_x_*) to probe the potential role of hydrogenation on weakening the N–N bond prior to activation. Scheme 1 summarizes the reaction network analyzed using DFT on the Fe(110) surface, including reactant adsorption, hydrogenation steps, and N–N activation pathways, along with the calculated effective enthalpy (Δ*H*҂) and free energy (Δ*G*҂) barriers (Equations (2) and (3)).

**Figure 2:**
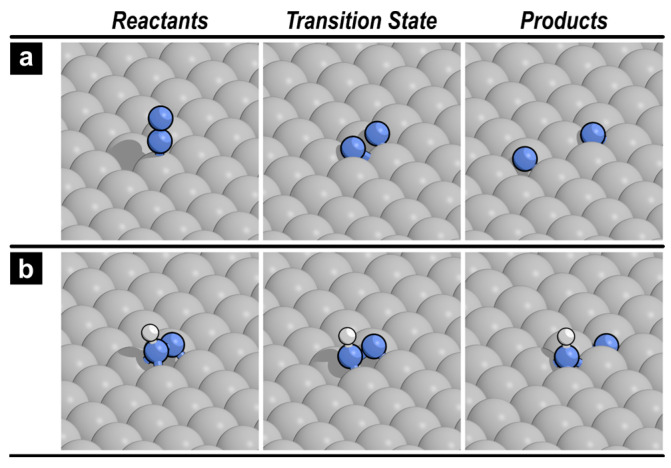
Reactants, transition state, and products’ structures for N–N bond cleavage in (**a**) *NN* and (**b**) *NNH* over the Fe(110) surface. Similar images for all other reactions on Fe(110) are depicted in Figure S1 in the Supplementary Materials.

The initial N_2_ adsorption on Fe(110) is calculated to be exothermic with an enthalpy of −40 kJ mol^−1^. Subsequent N–N bond cleavage can proceed either directly from adsorbed N_2_* (Figure 2a) or after partial hydrogenation to form the *NNH* intermediate (Figure 2b). While N–N bond activation in *NNH* has a lower enthalpic barrier (24 kJ mol^−1^; Scheme 1) than direct N_2_ dissociation (65 kJ mol^−1^), it is crucial to consider the influence of entropy on reaction rates. Since hydrogen adsorption from the gas phase leads to a decrease in entropy, free energy barriers are essential for comparing the feasibility of these pathways. Incorporating entropic effects reveals that the free energy barriers for N–N bond cleavage in both adsorbed N_2_* and *NNH* are calculated to be 174 kJ mol^−1^ (Scheme 1). However, *NNH* formation itself has a free energy barrier of 196 kJ mol^−1^, 22 kJ mol^−1^ higher than that of direct N_2_ dissociation. This suggests that, on Fe(110), direct N–N activation from the adsorbed N_2_* state is thermodynamically more favorable than a pathway involving initial hydrogenation.

**Figure 3:**
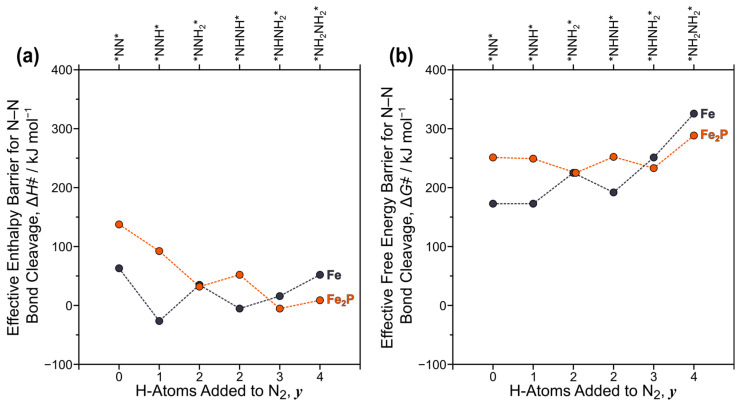
Effective (**a**) enthalpy (Δ*H*҂) and (**b**) free energy (Δ*G*҂) barriers for N–N bond activation in N_2_H*_x_* intermediates over Fe(110) and Fe_2_P(001) surfaces at 673 K.

After *NNH* is formed, it can undergo further hydrogenation to either *NNH_2_* or the isomeric form, diazene (*NHNH*). Diazene formation, however, is thermodynamically more favorable, with a free energy barrier of 258 kJ mol^−1^ compared to 272 kJ mol^−1^ for *NNH_2_* formation (Scheme 1). Moreover, N–N bond activation in diazene is more facile, requiring a free energy barrier of 193 kJ mol^−1^ compared to 226 kJ mol^−1^ for *NNH_2_* activation. Further hydrogenation steps from either intermediate (*NNH_2_* or *NHNH*) towards hydrazine (*NH_2_NH_2_*) involve substantial free energy barriers exceeding 300 kJ mol^−1^. Finally, N–N bond activation in *NHNH_2_ and *NH_2_NH_2_* requires free energy barriers of 252 kJ mol^−1^ and 326 kJ mol^−1^, respectively. These results highlight a general trend: while adding more hydrogen atoms to N_2_ progressively weakens the N–N bond enthalpically (Figure 3a), the accompanying entropy costs associated with adsorbing gas-phase hydrogen lead to higher free energy barriers for these later reaction steps (Figure 3b).

Previous experimental work revealed a reactivity trend for ammonia synthesis over different Fe surfaces: Fe(111) > Fe(100) > Fe(110), with Fe(110) being the least active under high pressure and temperature conditions (20 atm; 638–723 K) [20]. Here, the Fe(110) surface was chosen to establish a baseline, allowing the mechanistic and activation energy changes induced by phosphorus addition in Fe_2_P to be clearly discerned. Furthermore, work by Nørskov and collaborators highlighted the importance of steps and defects on Fe(110). It was shown that N–N activation is significantly more favorable on step sites (similar to those found on Fe(111)) compared to the flat surface [32]. These findings suggest that even on Fe(110), low-coordinated surface sites could play a crucial role in ammonia synthesis. Therefore, the modifications induced by the addition of phosphorus in Fe_2_P may potentially alter the surface structure and electronic properties, offering an opportunity for improved ammonia synthesis activity.

### 2.3. N–N Bond Activation Pathways on Fe_2_P(001)

**Scheme 2:**
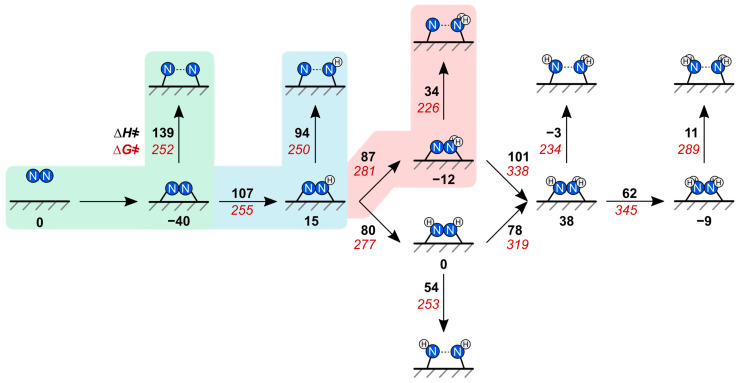
The examined reactions network on Fe_2_P(110) surface. Effective enthalpy (Δ*H*҂) and free energy (Δ*G*҂) barriers are shown in kJ mol^−1^ at 673 K, calculated relative to gas-phase N_2_ and H_2_ using Equations (2) and (3).

Building upon the understanding of N–N activation on the Fe(110) surface gained from the previous section, the corresponding mechanisms on the Fe_2_P(001) surface are analyzed in this section. This analysis aims to elucidate how phosphorus influences the energetics of N–N bond cleavage, identify potential changes in the preferred reaction pathways, and ultimately provide insights into the design of more active phosphide-based catalysts for ammonia synthesis. Scheme 2 shows the reaction pathways examined on the Fe_2_P(001) surface.

The initial N_2_ adsorption on Fe_2_P(001) remains exothermic, with an enthalpy similar to that on Fe(110) (−40 kJ mol^−1^). However, subsequent N–N bond cleavage pathways on Fe_2_P(001) diverge significantly from those observed on Fe(110). The direct N–N bond dissociation in adsorbed N_2_ has a higher enthalpic barrier on Fe_2_P(001) (Δ*H*҂ = 139 kJ mol^−1^) compared to Fe(110) (65 kJ mol^−1^). Activating the N–N bond in the *NNH* intermediate, however, has a significantly lower enthalpic barrier on Fe_2_P(001) (94 kJ mol^−1^). Importantly, when considering the entropic penalties associated with hydrogen adsorption, both activation routes on Fe_2_P(001) possess similar effective free energy (Δ*G*҂) barriers (approximately 250 kJ mol^−1^). The formation of *NNH* also has a free energy barrier of around 255 kJ mol^−1^ on Fe_2_P. Considering the expected uncertainty when comparing DFT-calculated energy barriers (typically around 5 kJ mol^−1^), this suggests that multiple competing N–N cleavage routes, direct N–N activation in N_2_ and the pathway involving *NNH* formation, might coexist on Fe_2_P(001).

**Figure 4:**
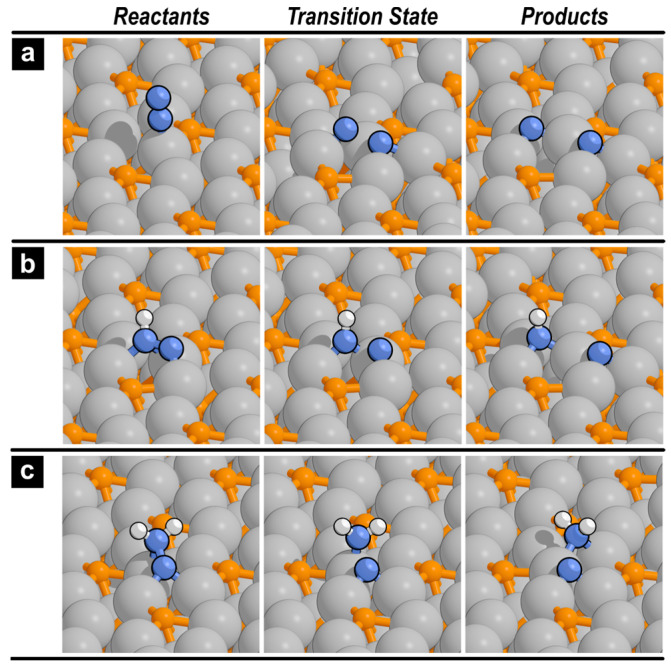
Reactants, transition state, and products’ structures for N–N bond cleavage in (**a**) *NN*, (**b**) *NNH*, and (**c**) *NNH_2_* over the Fe_2_P(001) surface. Similar images for all other reactions on Fe_2_P (001) are depicted in Figure S2 in the Supplementary Materials.

For direct N_2_ dissociation, the initial step involves N_2_ adsorption in a vertical atop configuration (Figure 4a), followed by a lateral shift to a parallel geometry. The transition state features both N atoms bridging two Fe atoms on the surface. Finally, N–N bond cleavage leads to adsorbed N atoms occupying separate three-fold (M_3_) sites. *NNH* activation, on the other hand, appears to proceed through a similar transition state structure on the same active site, with both N atoms bridging Fe atoms (Figure 4b). However, the presence of hydrogen weakens the N–N bond to a greater extent, leading to a lower enthalpic barrier for this pathway. The reaction products, N* and NH*, also occupy three-fold sites after the cleavage event. Here, phosphorus atoms do not directly participate in binding the transition states. Instead, their role lies in altering the electronic properties of the Fe atoms and also breaking up Fe atoms ensembles. The Fe–Fe bond distance in Fe_2_P(001) is larger (3.06 Å) compared to the Fe–Fe bond distances in the Fe(110) catalyst (2.48 Å). We have also shown previously that the incorporation of the phosphorus atom in Fe_2_P(001) results in a cationic shift in the surface Fe atoms to +0.09 e [27]. These modifications in the electronic structure and surface geometry induced by phosphorus likely contribute to the observed differences in the N–N activation pathways on Fe_2_P(001).

The N–N bond cleavage in diazene (*NHNH*) has a free energy barrier comparable to both direct N_2_* dissociation and *NNH* activation pathways (253 kJ mol^−1^), as shown in Scheme 2. While the *NNH_2_* pathway (Figure 4c) has a slightly lower N–N cleavage free energy barrier (226 kJ mol^−1^), its formation from *NNH* requires a significant barrier of 281 kJ mol^−1^. The familiar trade-off observed on Fe(110) is also seen here: hydrogenation can decrease enthalpic barriers for N–N cleavage, however, the entropic cost associated with hydrogen adsorption introduces higher overall free energy barriers. Further hydrogenation to *NHNH_2_* and hydrazine (*NH_2_NH_2_*) remains highly unfavorable due to their significant free energy barriers (>300 kJ mol^−1^).

These findings reveal that the introduction of phosphorus in Fe_2_P(001) leads to a more complex landscape for N–N bond activation compared to Fe(110). Unlike the single thermodynamically preferred pathway observed on Fe(110), Fe_2_P(001) exhibits multiple competitive N–N activation routes with similar free energy barriers. Phosphorus-induced modifications to the surface structure and electronic properties appear to be crucial factors in facilitating these alternative pathways. To further explore the implications of these findings, the next section investigates the turnover rates for these N–N activation routes, incorporating the effects of varying H_2_ pressure.

### 2.4. DFT-Predicted N–N Bond Cleavage Turnover Rate

Having established the energetics of N–N bond activation on both Fe(110) and Fe_2_P(001) surfaces, the focus now shifts to the prediction of the turnover rates for the competing pathways. Analyzing the influence of pressure on rate is important because ammonia synthesis typically occurs at high pressure and temperature conditions. Using the DFT-derived free energy barriers at 673 K in conjunction with Equation (4), it is possible to assess how these rates depend on both N_2_ and H_2_ pressures, providing insights into the feasibility of each pathway under practical NH_3_ synthesis conditions. Analysis of Figure 3b reveals that, on Fe(110), direct N_2_ dissociation and *NNH* activation have similar free energy barriers (174 kJ mol^−1^). Moreover, Fe_2_P(001) exhibits multiple competing N–N cleavage routes with close free energy barriers (226–255 kJ mol^−1^). This finding highlights the need to examine the N–N activation rate as a function of H_2_ pressure, as the pathways involving pre-hydrogenation could become more favorable at higher H_2_ pressures according to Equation (4).

**Figure 5:**
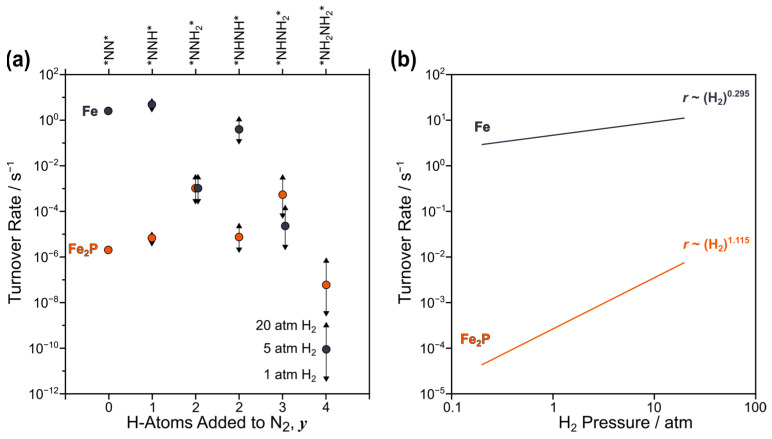
(**a**) DFT-predicted rates for N–N bond cleavage in N_2_H*_x_* intermediates (5 atm H_2_, 5 atm N_2_, 673 K). Up/down arrows represent 20 and 1 atm H_2_, respectively. (**b**) Total N–N bond activation rate as a function of H_2_ pressure (5 atm N_2_, 673 K).

Figure 5a illustrates the DFT-predicted N–N bond cleavage turnover rates for all N_2_H*_x_* intermediates on Fe(110) and Fe_2_P(001) at 5 atm H_2_ pressure, with arrows indicating how the rates change between 1 atm and 20 atm H_2_. For more hydrogenated intermediates, larger arrows emphasize their greater sensitivity to H_2_ pressure. On Fe(110), *NNH* activation exhibits the highest rate (4.52 s^−1^) at 5 atm H_2_, followed closely by direct N_2_ dissociation (2.39 s^−1^). All other intermediates show significantly lower rates, even at 20 atm H_2_. However, as shown in Scheme 1, the high free energy barrier (196 kJ mol^−1^) for *NNH* formation makes this pathway less likely compared to direct N_2_ dissociation (174 kJ mol^−1^). These results are consistent with experiments [20,21], where Fe(111) exhibits a higher turnover rate (9.7 s^−1^) at 15 atm H_2_ and 5 atm N_2_ compared to the predicted 2.39 s^−1^ value here for the less reactive Fe(110) surface.

For Fe_2_P(001), Figure 5a shows that N–N bond cleavage in *NNH_2_* has the highest calculated turnover rate (1.02 × 10^−3^ s^−1^) at 5 atm H_2_, followed closely by *NHNH_2_* (5.21 × 10^−4^ s^−1^). However, the large free energy barriers for forming *NNH_2_*, *NHNH*, and *NHNH_2_*, as shown in Scheme 2, likely limit their overall contribution to ammonia synthesis. A crucial difference from Fe(110) emerges here where N–N bond cleavage in *NNH* on Fe_2_P(001) exhibits a higher turnover rate (6.37 × 10^−6^ s^−1^) than direct N_2_ dissociation (1.93 × 10^−6^ s^−1^). Furthermore, Scheme 2 reveals that the formation of *NNH* on Fe_2_P(001) has a free energy barrier comparable to direct N_2_ dissociation. This suggests that N–N bond activation via the *NNH* intermediate could be a significant pathway on Fe_2_P(001), in contrast to Fe(110), where direct N_2_ dissociation likely dominates.

Figure 5b reveals a clear distinction between Fe(110) and Fe_2_P(001) in the total N–N activation rate (sum of turnover rates for N–N bond activation in all N_2_H*_x_* intermediates) dependence on H_2_ pressure. On Fe(110), the total N–N activation rate exhibits a H_2_ pressure dependence of approximately [H_2_]^0.295^ (Equation (4)), indicating a greater contribution from intermediates with fewer hydrogen atoms. In contrast, Fe_2_P(001) displays a much stronger dependence ([H_2_]^1.115^), suggesting that more hydrogenated N_2_H*_x_* species are likely crucial for the ammonia synthesis rate. This fundamental difference highlights the potential of phosphide-based catalysts to unlock alternative ammonia synthesis mechanisms. This shift towards activation in more hydrogenated species, while maintaining favorable NH*_x_* binding energies, could be an important design consideration for developing improved ammonia synthesis catalysts.

## 3. Computational Methods

All periodic DFT calculations were carried out using the Vienna ab initio simulation package (VASP) [33,34,35,36], in conjunction with the computational catalysis interface (CCI) [37]. The projector augmented-wave (PAW) method was employed with a plane-wave energy cutoff of 396 eV [38,39]. Exchange-correlation energies were treated using the revised Perdew−Burke−Ernzerhof (RPBE) functional [40,41,42]. Gaseous species were modeled using 15 × 15 × 15 Å unit cells. The initial bulk structures for Fe (space group *Im*$\bar{3}$*m*) and Fe_2_P (space group *P*$\bar{6}2$*m*) were obtained from crystallographic data [43,44]. DFT optimization was then performed to determine lattice parameters, resulting in values of (a = b = c = 2.83 Å) for Fe and (a = b = 5.81 Å, c = 3.42 Å) for Fe_2_P. Spin-polarization was included in all calculations due to the ferromagnetic nature of Fe and Fe_2_P.

**Figure 6:**
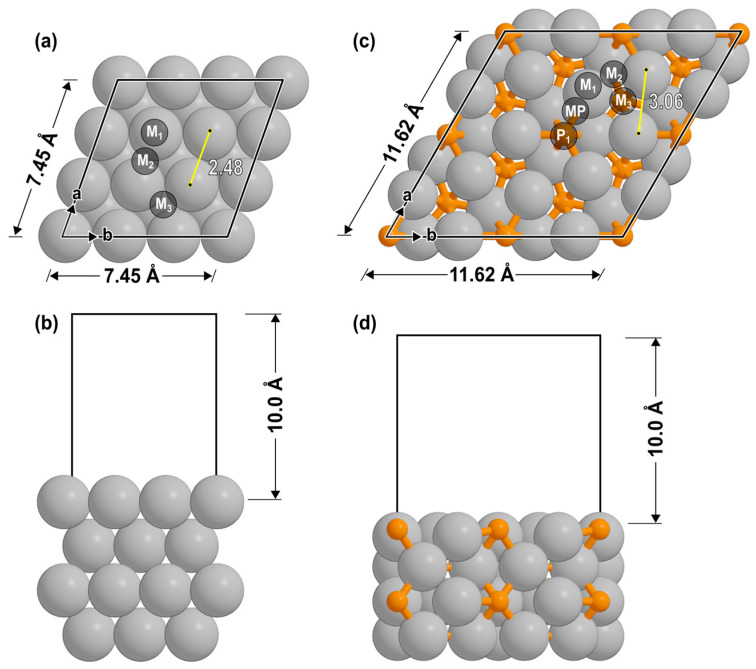
(**a**,**b**) Fe(110) and (**c**,**d**) Fe_2_P(001) slabs as seen from top and side views. Labels denote the binding sites (M_1_: metal atop; M_2_: metal–metal bridge; M_3_: metal three-fold hollow; MP: metal–phosphorus bridge; P_1_: phosphorus atop).

The closed-packed Fe(110) surface was modeled using a 3 × 3 periodic lattice with four atomic layers and 10 Å of vacuum orthogonal to the surface (Figure 6a,b). For the Fe_2_P catalyst, the Fe-rich (001) surface (Figure 6c), which is identical to the Ni_2_P(001) surface previously shown to be active for selective C–O bond activation [24], was constructed using two repeating units (four atomic layers) and a 10 Å vacuum layer (Figure 6d). In both models, the bottom two layers were fixed during optimization and a *k*-point mesh of 3 × 3 × 1 was used for all surface calculations [45,46].

Transition state structures were located using a combination of the nudged elastic band (NEB) method and the dimer method [47,48,49]. The convergence criteria for electronic energies and forces were set to 10^−6^ eV and 0.05 eVÅ^−1^, respectively. Frequency calculations were performed, with all catalysts’ atoms constrained, to estimate enthalpies and free energies at 673 K. The binding energy (Δ*E_ads_*) is defined as:

(1) $$∆E_{ads}=E_{species/surf\;}-E_{species(g)}-E_{surf}$$

and effective enthalpy (Δ*H*҂) and free energy (Δ*G*҂) barriers are calculated using:

(2) $$∆H҂=H҂-0.5yH_{H2(g)}-H_{N2(g)}-H_{surf}$$

(3) $$∆G҂=G҂-0.5yG_{H2(g)}-G_{N2(g)}-G_{surf}$$

at 673 K where *y* is the number of hydrogen atoms added to N_2_ prior to N–N activation. Since ammonia synthesis typically occurs at high pressure conditions, it is essential to analyze the influence of pressure on the turnover rate using the DFT-derived free energy barriers. The N–N bond cleavage turnover rate can be predicted by:

(4) $$\frac{r}{\left[L\right]}=\frac{k_{B}T}{h}exp\left(\frac{-G҂}{RT}\right)\left(N_{2}\right){\left(H_{2}\right)}^{0.5y}$$

assuming that the *N*_2_ hydrogenation steps are quasi-equilibrated and N–N activation is the rate-limiting step. More details of the computational methods can be found in Section S1 in the Supplementary Materials.

## 4. Conclusions

In this study, the mechanisms of ammonia synthesis on Fe(110) and Fe_2_P(001) surfaces have been systematically examined. The results illustrate that the incorporation of phosphorus significantly alters catalyst behavior. The weakening of NH*_x_* intermediate binding on Fe_2_P(001) suggests the potential for more facile hydrogenation steps compared to pure Fe(110). We also find that multiple competing N–N activation pathways become viable on Fe_2_P(001), including the routes involving pre-hydrogenated intermediates like *NNH*. Moreover, analysis of the H_2_ pressure dependence of turnover rates reveals that contributions from more hydrogenated intermediates could become dominant on Fe_2_P(001), unlike on Fe(110), where direct N_2_ dissociation remains the primary pathway. 

This shift away from the single pathway limitation of traditional transition metal catalysts offers a promising avenue for catalyst design. Phosphorus-modified catalysts could potentially circumvent the limitations imposed by the traditional BEP relationship, allowing the design of highly active catalysts operating under milder conditions. The theoretical insights provided in this work lay a foundation for the rational design of Fe-based phosphide catalysts tailored for improved ammonia synthesis performance. Further studies, including investigations of the influence of phosphorus content and the potential role of step sites on Fe_2_P, will be crucial for realizing the full potential of phosphide-based materials for sustainable ammonia production.

## Figures and Tables

**Table 1:** Binding energies Δ*E*_ads_ (Equation (1); kJ mol^−1^) of examined intermediates and their preferred adsorption configuration on Fe(110) and Fe_2_P(001).

	Fe(110)		Fe_2_P(001)	
Species	Adsorption Mode	Δ*E*_ads_	Adsorption Mode	Δ*E*_ads_
		kJ mol^−1^		kJ mol^−1^
N_2_*	M_1_	−33	M_1_	−47
N*	M_3_	−576	M_3_	−518
NH*	M_3_	−465	M_3_	−426
NH_2_*	M_2_	−263	M_2_	−258
NH_3_*	M_1_	−52	M_1_	−64
H*	M_3_	−272	M_3_	−256

## Data Availability

Dataset available on request from the author.

## Supplementary Materials

The following supporting information can be downloaded at: https://www.mdpi.com/article/10.3390/molecules29081894/s1, Section S1: Computational Methods Details; Figure S1: Reactions images on Fe(110) surface; Figure S2: Reactions images on Fe_2_P(001) surface. References [43,44,50] are cited in the Supplementary materials.
