# Supplementary material for: Phosphorus Modification of Iron: Mechanistic Insights into Ammonia Synthesis on Fe2P Catalyst
Source: Molecules. 2024 Apr 22;29(8):1894. doi: 10.3390/molecules29081894 (PMC11054198; doi:10.3390/molecules29081894)
Supplement: Supplementary file 1 [file molecules-29-01894-s001.zip › molecules-2966076-supplementary.pdf]

molecules

Supplementary Materials

Phosphorus Modification of Iron: Mechanistic Insights into Ammonia Synthesis on Fe_2P Catalyst

Abdulrahman Almithn

Department of Chemical Engineering, College of Engineering, King Faisal University, Al Ahsa 31982, Saudi Arabia; aalmithn@kfu.edu.sa

S1. Computation Methods Details

The initial bulk structures for Fe (space group $Im\bar{3}m$) and Fe_2P (space group $P\bar{6}2m$) were obtained from crystallographic data [43,44]. DFT optimization was then performed to determine lattice parameters, resulting in values of (a = b = c = 2.83 Å) for Fe and (a = b = 5.81 Å, c = 3.42 Å) for Fe_2P. Spin polarization was included in all calculations due to the ferromagnetic nature of Fe and Fe_2P.

Frequency calculations were performed on gas-phase molecules and all optimized adsorbed species to determine zero-point vibrational energies (ZPVE) and vibrational, translational, and rotational enthalpy and free energy. These terms were then used, together with electronic energies (E_0, provided by VASP), to estimate enthalpies (H)

$$H = E_0 + \text{ZPVE} + H_{\text{vib}} + H_{\text{trans}} + H_{\text{rot}} \quad \text{(S1)}$$

and free energies (G)

$$G = E_0 + \text{ZPVE} + G_{\text{vib}} + G_{\text{trans}} + G_{\text{rot}} \quad \text{(S2)}$$

for reactants, products, and transition states at 673 K (a typical temperature for NH_3 synthesis). For calculations which include a periodic metal surface, there are no translational or rotational degrees of freedom, and DFT-derived vibrational frequencies can be used to determine the ZPVE, H_{vib}, and G_{vib}

$$ZPVE = \sum_i (½\nu_i h) \quad \text{(S3)}$$

$$H_{vib} = \sum_i \left(\frac{\nu_i h e^{\frac{-\nu_i h}{kT}}}{1-e^{\frac{-\nu_i h}{kT}}} \right) \quad \text{(S4)}$$

$$G_{vib} = \sum_i \left(-kT \ln \frac{1}{1-e^{\frac{-\nu_i h}{kT}}} \right) \quad \text{(S5)}$$

For gaseous molecules, translational and rotational enthalpies and free energies were also computed from statistical mechanics

$$H_{trans} = \frac{5}{2} kT \quad \text{(S6)}$$

$$H_{rot,linear} = kT \quad \text{(S7)}$$

$$H_{rot,nonlinear} = \frac{3}{2} kT \quad \text{(S8)}$$

$$G_{trans} = -kT \ln[\left(\frac{2\pi MkT}{h^2}\right)^{3/2} V] \quad \text{(S9)}$$

$$G_{rot} = -kT \ln[\frac{\pi^{1/2}}{\sigma}\left(\frac{T^3}{\theta_x \theta_y \theta_z}\right)^{1/2}] \quad \text{(S10)}$$

$$\theta_i = \frac{h^2}{8\pi^2 I_i k} \quad \text{(S11)}$$

where I_i is the moment of inertia about axes x, y, or z and σ is the symmetry number of the molecule (2 for H_2 and 6 for C_2H_6). Equations S6–S10 were obtained from: McQuarrie, D. A.; Statistical Mechanics; Sausalito, CA [50].

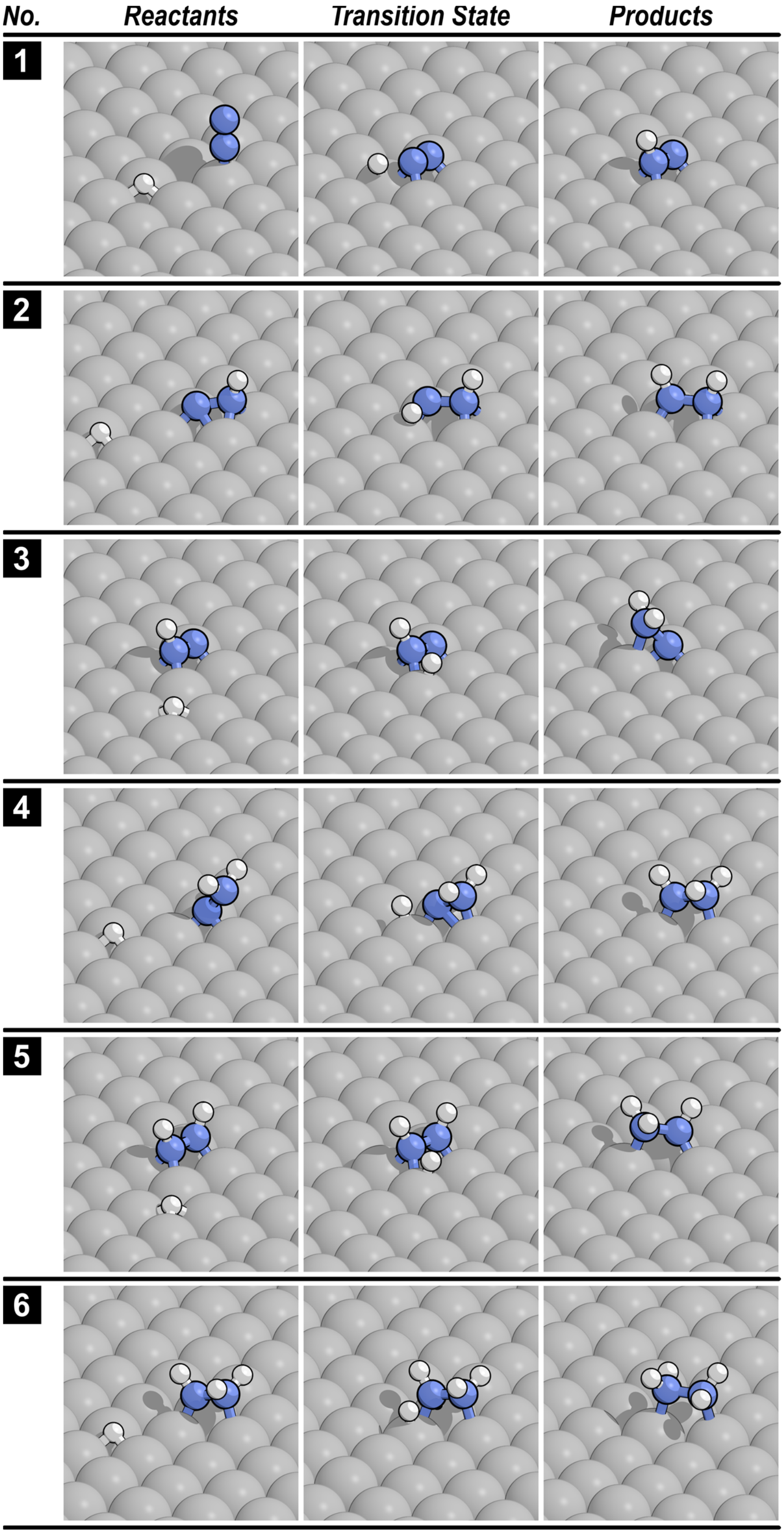

Figure S1. Reactants, transition state, and products' structures for all reactions examined over Fe(110) surface.

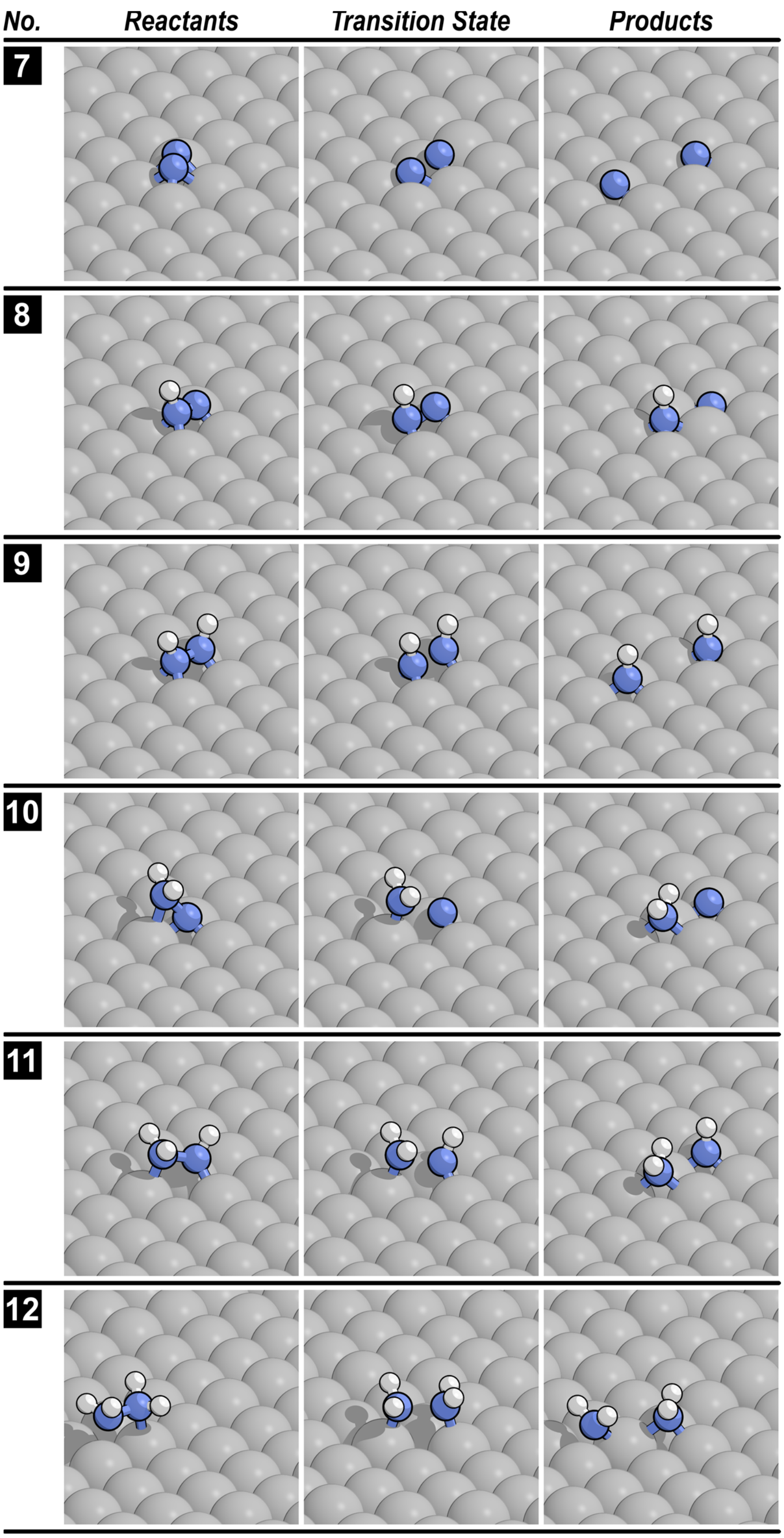

Figure S1 Cont. Reactants, transition state, and products' structures for all reactions examined over Fe(110) surface.

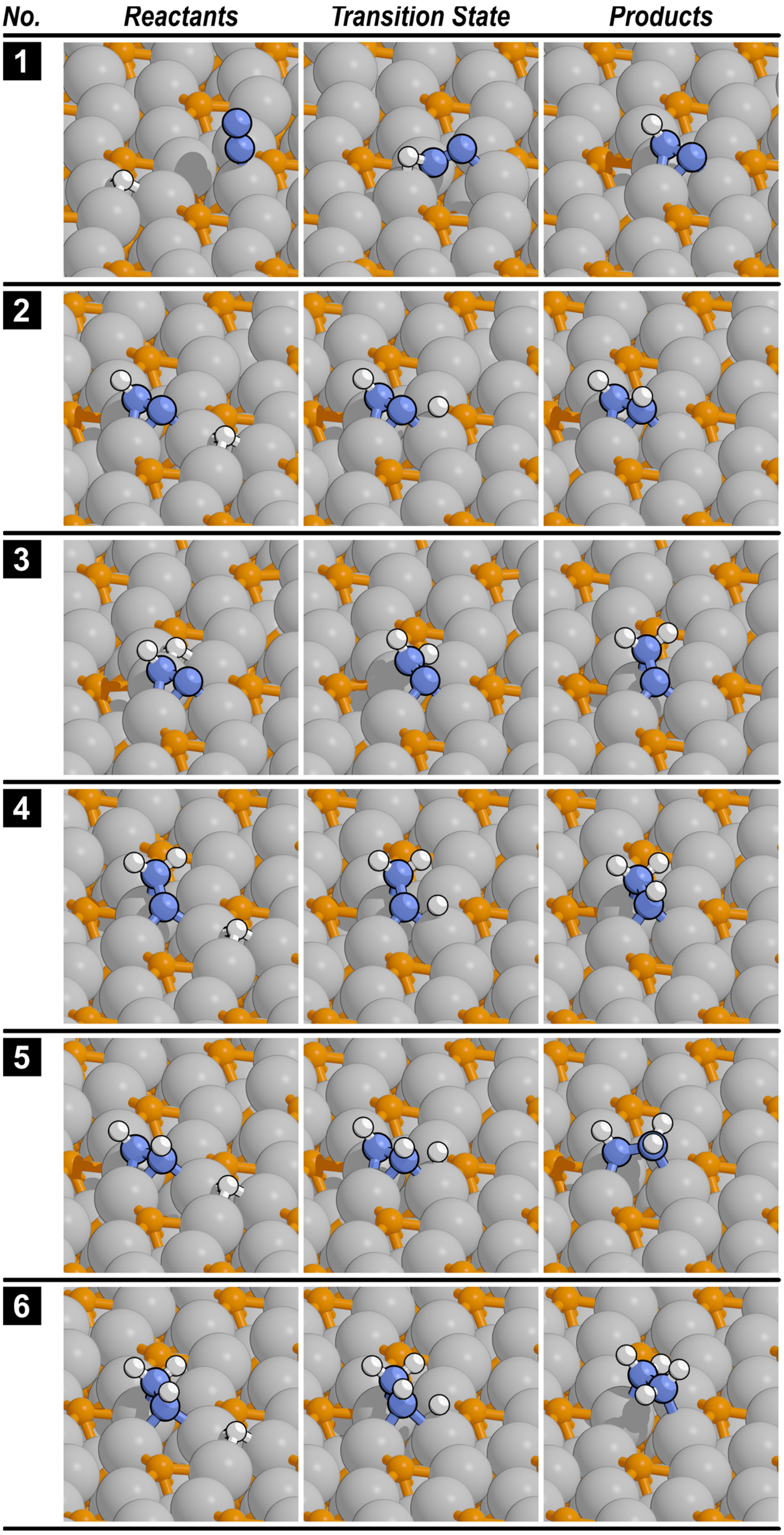

Figure S2. Reactants, transition state, and products' structures for all reactions examined over $Fe_2P(001)$ surface.

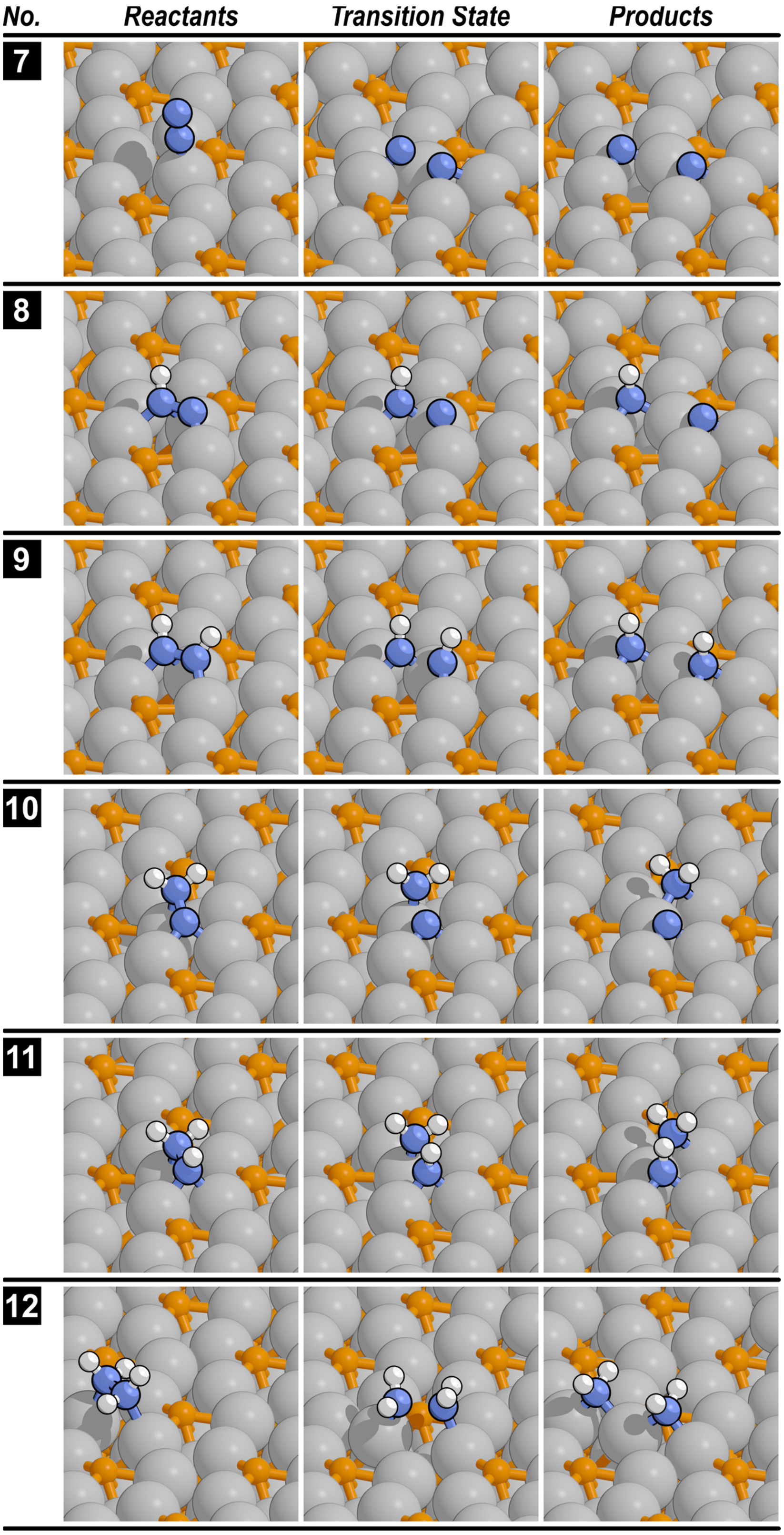

Figure S2 Cont. Reactants, transition state, and products' structures for all reactions examined over $Fe_2P(001)$ surface.

References

43. Wilburn, D.R.; Bassett, W.A. Hydrostatic Compression of Iron and Related Compounds; an Overview. *Am. Mineral.* **1978**, *63*, 591–596.

44. Koumina, A.; Bacmann, M.; Fruchart, D.; Soubeyroux, J.-L.; Wolfers, P.; Tobola, J.; Kaprzyk, S.; Niziol, S.; Mesnaoui, M.; Zach, R. Crystallographic and Magnetic Properties of Fe_2P. *Ann. Chim. Sci. Des Mater.* **1998**, *23*, 177–180.

50. McQuarrie, D.A. Statistical Mechanics; University Science Books: Sausalito, CA, USA, 2000.
